# Comparison of Efficacy and Safety of Angiotensin Receptor-Neprilysin Inhibitors in Patients With Heart Failure With Reduced Ejection Fraction: A Meta-Analysis

**DOI:** 10.7759/cureus.36392

**Published:** 2023-03-20

**Authors:** Muhammad Talha Haseeb, Muhammad Nouman Aslam, FNU Avanteeka, Umar Abdul Rehman Khalid, Dewan Zubaer Ahmad, Mithum Senaratne, Bsher Almaalouli, Shamsha Hirani

**Affiliations:** 1 Department of Internal Medicine, Shaikh Zayed Hospital, Lahore, PAK; 2 College of Medicine, King Edwards Medical University, Lahore, PAK; 3 Internal Medicine, Liaquat University of Medical and Health Sciences, Jamshoro, PAK; 4 Internal Medicine, King Edward Medical College, Lahore, PAK; 5 Medicine, University of Dhaka, Dhaka, BGD; 6 Medicine, University of Ruhana, Matara, LKA; 7 Faculty of Medicine, Damascus University, Damascus, SYR; 8 Cardiology, Baqai Hospital, Karachi, PAK

**Keywords:** meta-analysis, efficacy, safety, heart failure with reduced ejection fraction, angiotensin receptor-neprilysin inhibitors

## Abstract

The present meta-analysis was conducted to compare the safety and efficacy of angiontensin receptor neprilysin inhibitor (ARNI) with angiotensin receptor blockers (ARBs) and angiotensin-converting-enzyme inhibitors (ACEi) in patients with heart failure with reduced ejection fraction (HFrEF). This meta-analysis was conducted and reported in accordance with the guidelines of the Preferred Reporting Items for Systematic Reviews and Meta-analysis (PRISMA) statement. Two authors carried out a scientific literature search on online databases, including EMBASE, PubMed, and the Cochrane Library. The following keywords or corresponding Medical Subject Headings (MeSH) were used for the search of relevant articles: “heart failure with reduced ejection fraction,” “angiotensin receptor-neprilysin inhibitor,” “Angiotensin receptor blockers,” and “clinical outcomes.” Outcomes assessed in the present meta-analysis included changes in ejection fraction (EF) from baseline in percentage. Other outcomes assessed in the present meta-analysis included all-cause mortality, cardiovascular death, and hospitalization due to heart failure. Adverse events assessed in the present meta-analysis included hypokalemia, acute kidney injury, and hypotension. Total 10 studies were included. This meta-analysis showed that treatment with ARNI was associated with a significantly lower risk of all-cause mortality and cardiovascular death compared to control groups. There was no significant difference between the two groups in terms of change of EF from baseline or hospitalization related to heart failure. However, the risk of hypotension was significantly higher in patients receiving ARNI. The study findings support the use of ARNI as first-line therapy for heart failure with reduced ejection fraction. Further studies are required to determine the optimal use of ARNI in heart failure management and to investigate the mechanisms underlying the increased risk of hypotension.

## Introduction and background

Heart failure is a clinical condition resulting from various cardiac diseases. In recent years, the incidence of heart failure has been consistently increasing and is predicted to continue due to the ageing population with more cardiovascular risk factors [1-2]. Heart failure with reduced ejection fraction (HFrEF) is responsible for 50% of the cases and is complicated by significant morbidity and mortality [3]. Apart from high morbidity and mortality rates, heart failure is consistently ranked as the most expensive condition in the United States, with costs amounting to $30.7 billion, two-thirds of which is due to direct medical expenses [4]. The high rate of 30-day rehospitalization among Medicare beneficiaries with heart failure, which stands at 18.2%, places a heavy cost burden on acute-care facilities [5].

After the publication of the Prospective comparison of angiotensin receptor-neprilysin inhibitor (ARNI) with angiotensin-converting enzyme inhibitor (ACEI) to determine the Impact on Global Mortality and morbidity in Heart Failure (PARADIGM-HF) trial [6], sacubitril/valsartan, an angiotensin receptor-neprilysin inhibitor (ARNI), was added to the guidelines for treating HFrEF. The analysis found that sacubitril/valsartan was more effective in decreasing hospitalization and cardiovascular mortality compared to enalapril [6]. Based on this evidence, ARNI has been approved for the treatment of HFrEF in more than 100 countries and is recommended by guidelines as a replacement for ARBs (angiotensin receptor blockers) or ACEi to decrease mortality and morbidity in patients with chronic HFrEF [7-8]. In addition, based on studies from the PIONEER HF trial and Transition trial [9-10], the European Society of Cardiology and American College of Cardiology expert consensus statements recommend the initiation of ARNi for patients hospitalized with new-onset heart failure or decompensated chronic heart failure with or without prior exposure to ACEi or ARBs [11].

Since the PARADIGM-HF trial, several new trials and retrospective studies on the efficacy and safety of ARNI trials have been conducted, including the PARALLEL-HF trial [12] and the OUTSTEP-HF trial [13], which did not report a reduction in certain outcomes, including length of hospital stay, number of days, and reduction in heart failure events among patients with HFrEF. Considering the new studies conducted, we performed the present meta-analysis to compare the safety and efficacy of ARNI with ARBs and ACEi in patients with HFrEF.

## Review

Methodology

This meta-analysis was conducted and reported in accordance with the guidelines of the Preferred Reporting Items for Systematic Reviews and Meta-analysis (PRISMA) statement.

Literature Search

Two authors carried out a scientific literature search on online databases, including EMBASE, PubMed, and the Cochrane Library. The following keywords or corresponding Medical Subject Headings (MeSH) were used for the search of relevant articles: “heart failure with reduced ejection fraction,” “angiotensin receptor-neprilysin inhibitor,” “Angiotensin receptor blockers,” and “clinical outcomes.” We also manually searched the reference lists of the included studies and reviews. The search was limited to articles published in English, and the time limit was from inception to February 1st, 2023.

Literature Screening and Data Extraction

Two researchers independently screened the literature and extracted data. First, title and abstract screening were conducted after removing duplicates. Full texts of all eligible studies were retrieved and screened for eligibility criteria using pre-specified inclusion and exclusion criteria. Disagreements between the two researchers were resolved through discussion. Data were extracted using pre-designed data extraction forms created using Microsoft Excel. Data extracted included the author's name, year of publication, study design, study groups, sample size, follow-up duration, and participants’ characteristics.

Eligibility Criteria and Measurement of Outcomes

We included randomized control trials (RCTs) and retrospective studies comparing ARNI with ARB or ACEi in patients with HFrEF. The inclusion criteria were as follows: (a) RCTs or retrospective studies, (b) patients with HFrEF with left ventricular EF of <45%. We excluded studies that did not report outcomes in the present meta-analysis. We excluded case reports, case series, reviews, and editorials. We also excluded studies that lacked a comparison group.

Outcomes assessed in the present meta-analysis included changes in ejection fraction (EF) from baseline in percentage. Other outcomes assessed in the present meta-analysis included all-cause mortality, cardiovascular death, and hospitalization due to heart failure. Adverse events assessed in the present meta-analysis included hypokalemia, acute kidney injury, and hypotension (as defined by individual studies).

Risk of Bias Assessment

Risk of bias assessment of each included study was assessed by two authors independently using the Cochrane Risk of Bias Assessment tool. Any disagreements between two authors were resolved by consensus and discussion. Seven domains were assessed and each domain was rated as high risk, low risk or unclear risk of bias as per the judgment criteria. For retrospective studies, Newcastle-Ottawa Quality Assessment was used. 

Statistical Analysis

We used Review Manager 5.4.1 software (The Cochrane Collaboration, Oxford, United Kingdom) for data analysis. The heterogeneity among the study results was assessed using I-square statistics. An I-square value of less than or equal to 50% indicated low heterogeneity among the study results, and a fixed-effect model was used for data analysis. In cases of heterogeneity of more than 50%, a random-effect model was used. Categorical outcomes were expressed as risk ratio (RR) and 95% confidence interval (CI), while continuous variables were presented as mean difference (MD) with their 95% CI. In the present meta-analysis, a p-value of 0.05 was used as a cut-off. Subgroup analysis was also performed on the basis of study design. 

Results

**Figure 1:**
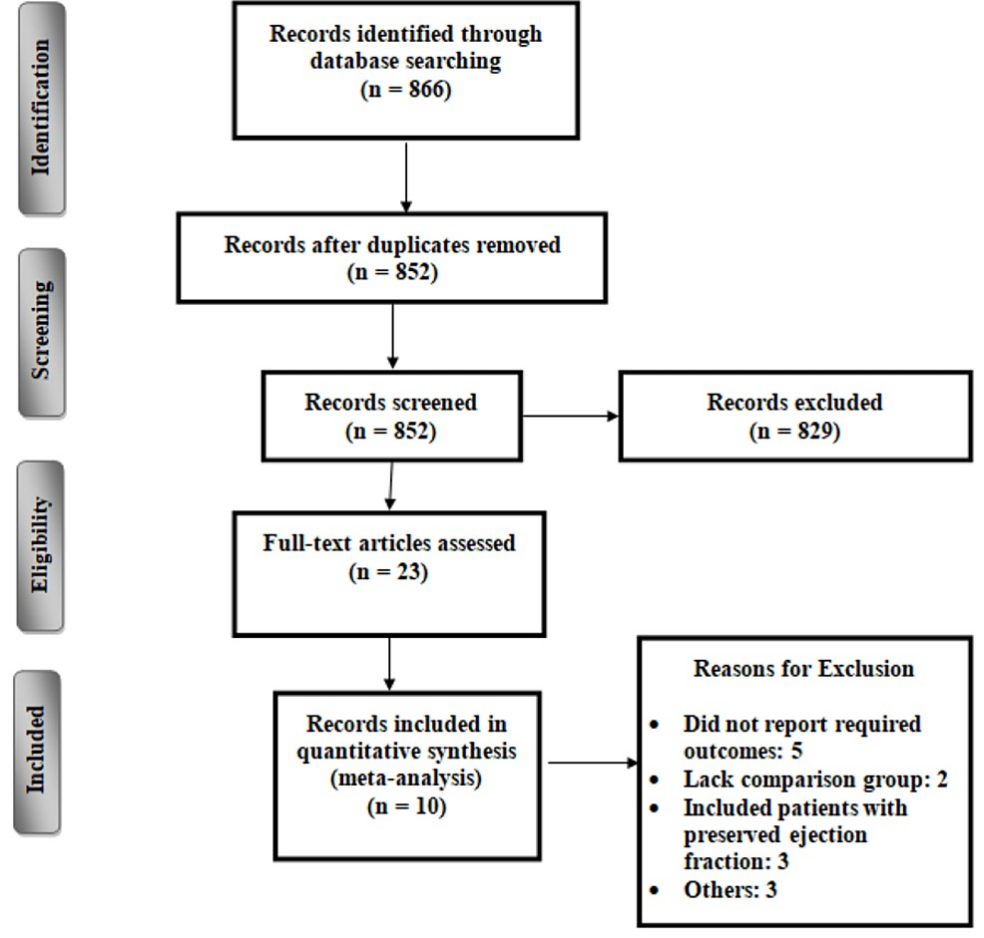
PRISMA flowchart of selection of studies

**Figure 2:**
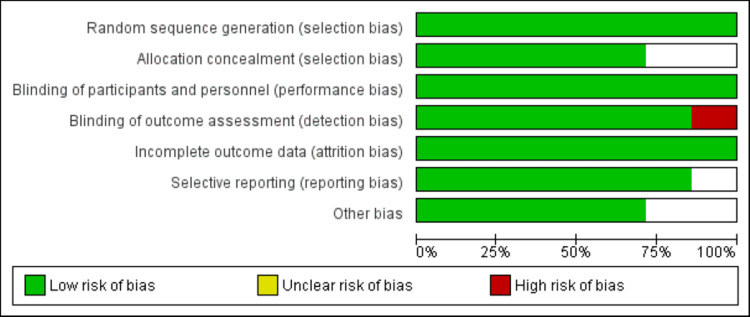
Risk of bias graph for randomized control trials (RCTs)

The process of study selection is presented in Figure 1. We identified 866 articles through database searching. We excluded 829 articles based on titles and abstract screening. After reviewing full texts of 23 articles, we further excluded 13 studies based on the pre-specified inclusion and exclusion criteria. Eventually, 10 studies were included in the current meta-analysis. Table 1 shows the characteristics of the included studies. Out of 10 studies, seven were RCTs, while three were retrospective studies. In all included studies, majority of the patients were male. The follow-up of included studies ranged from eight weeks to 96 weeks. Figure 2 shows risk of bias assessment of included RCTs. Among all RCTs, six were double-blinded and one was single blinded study. Overall risk of bias was low. Table 2 shows quality assessment of cohort studies. Two studies were having good quality based on Newcastle-Ottawa scales.

**Table 1:** Characteristics of included studies ARNI: Angiontensin Receptor Neprilysin Inhibitor; ARB: Angiotensin receptor blockers; ACEI: Angiotensin-converting-enzyme inhibitors; RCT: Randomized control trials; NR: Not reported

Author	Year	Design	Groups	Comparison Group	Sample Size	Follow-up	Mean Age (Years)	Male (%)
Atan et al. [14]	2022	Retrospective	ARNI		40	18 Weeks	NR	NR
			Control	ACEI	40			
Chapman et al. [15]	2022	Retrospective	ARNI		758	96 Weeks	63.9 vs 66.4	71 vs 75
			Control	ACEI/ARB	758			
Desai et al. [16]	2019	RCT	ARNI		231	24 Weeks	67.8 vs 66.7	74 vs 79
			Control	ACEI	233			
Ghafur et al. [17]	2020	RCT	ARNI		50	12 Weeks	60.8 vs 61.9	66 vs 68
			Control	ARB	50			
Hsiao et al. [18]	2022	Retrospective	ARNI		206	12 Weeks	65.1 vs 66.8	68.9 vs 67.1
			Control	ACEI/ARB	833			
Kang et al. [19]	2019	RCT	ARNI		51	36 Weeks	60.5 vs 64.7	65.5 vs 56.7
			Control	ARB	53			
McMurry et al. [6]	2014	RCT	ARNI		4203	8 Weeks	63.8 vs 63.8	79 vs 77.4
			Control	ACEI	4229			
Piepoli et al. [13]	2021	RCT	ARNI		309	12 Weeks	67.16 vs 66.6	77.02 vs 80.32
			Control	ACEI	310			
Tsutsui et al. [12]	2021	RCT	ARNI		111	24 Weeks	69.0 vs 66.7	86.5 vs 85.7
			Control	ACEI	112			
Velazquez et al. [9]	2019	RCT	ARNI		440	8 Weeks	61 vs 63	74.3 vs 69.8
			Control	ACEI	441			

**Table 2:** Quality assessment of cohort studies

Study ID	Selection	Comparibility	Outcome	Total
Atan et al. [14]	3	1	2	Good
Chapman et al. [15]	4	1	2	Fair
Hsiao et al. [18]	4	1	2	Good

Change of Ejection Fraction (EF) from Baseline (%)

**Figure 3:**
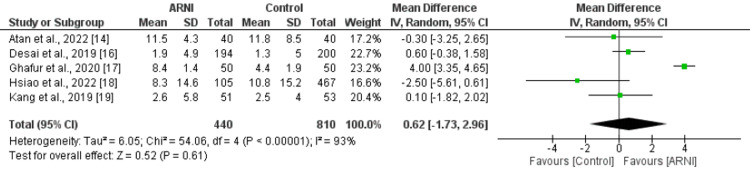
Forest plot comparing change of ejection fraction from baseline between ARNI group and control group. Sources: References [14,16-19] ARNI: Angiontensin Receptor Neprilysin Inhibitor

Five studies that compared the change of EF from baseline between patients who received ARNI and patients in the control group reported no significant differences between the groups in the change of EF from baseline. The mean difference was 0.62, with a 95% confidence interval of -1.73 to 2.96 and a p-value of 0.61. However, high heterogeneity was reported among the study results, with an I-square of 93% as shown in Figure 3. We conducted subgroup analysis on the basis of study design. Both retrospective and prospective studies showed similar results compared to the overall pooled analysis as shown in Table 2. However, heterogeneity was reduced at from 91% to 71%.

All-Cause Mortality and Cardiovascular Mortality

**Figure 4:**
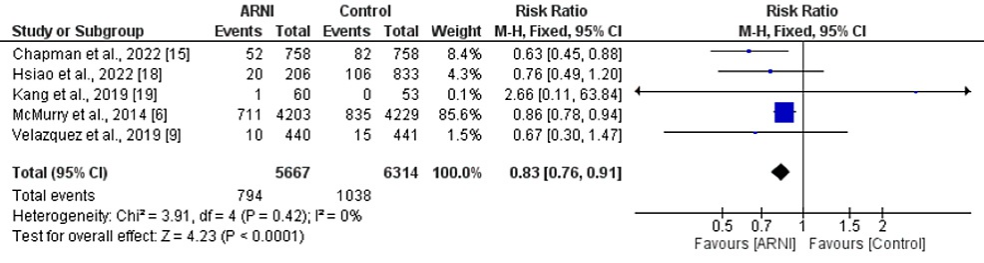
Forest plot comparing all-cause mortality between ARNI group and control group ARNI: Angiontensin Receptor Neprilysin Inhibitor Sources: References [6,9,15,18,19]

A total of five studies were included in the analysis of the all-cause mortality outcome, comparing ARNI versus control. A fixed effect model was used to estimate the effect size. The results showed that 794 out of 5,667 patients who received ARNI experienced an event, compared to 1,038 out of 6314 patients in the control group. The relative risk (RR) of all-cause mortality was 0.83 (95% CI: 0.76, 0.91, p-value<0.001), indicating that patients who received ARNI had a significantly lower risk of all-cause mortality compared to those in the control group as shown in Figure 4. There was no heterogeneity among the studies, as indicated by an I-square of 0%. With subgroup analysis both retrospective and prospective studies showed similar results compared to the overall pooled analysis as shown in Table 2.

**Figure 5:**
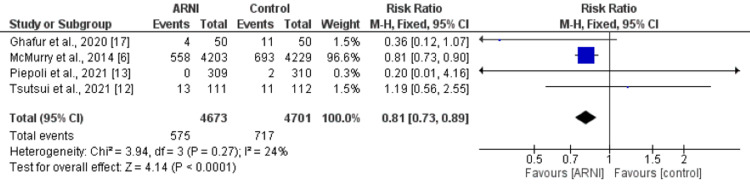
Forest plot comparing cardiovascular death between ARNI group and control group ARNI: Angiontensin Receptor Neprilysin Inhibitor Sources: References [6,12,13,17]

Treatment with an ARNI was associated with significantly lower risk of cardiovascular death (RR: 0.81, 95%: 0.73, 0.89, p-value<0.001). Low heterogeneity was reported among the study results (I-square: 24%) as shown in Figure 5.

Hospitalization Related to Heart Failure

**Figure 6:**
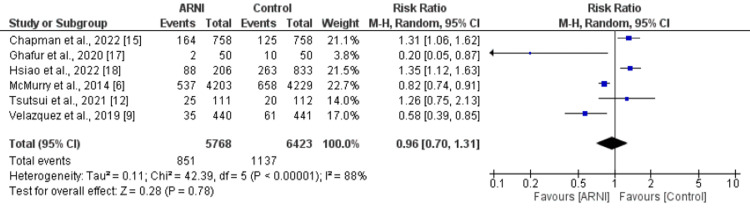
Forest plot comparing hospitalization due to heart failure between ARNI group and control group ARNI: Angiontensin Receptor Neprilysin Inhibitor Sources: References [6,9,12,15,17-18]

Six studies assessed the impact of ARNI on hospitalization. The meta-analysis reported no significant difference between patients in ARNI group and patients in control group in relation to risk of hospitalization (RR: 0.96, 95% CI: 0.70, 1.31, p-value: 0.78) as shown in Figure 6. High heterogeneity was reported among the study results (I-square: 88%). Pooled analysis of RCTs showed risk of hospitalization was low in patients receiving ARNI but the difference was statistically insignificant. However, retrospective studies pooled analysis showed higher risk of hospitalization in control group as shown in Table 3. 

**Table 3:** Results of subgroup analysis * Presented as mean difference (95% CI) ^ Significant at p-value<0.05 (Favors ARNI) RR: Risk ratio, CI: Confidence Interval

Outcome	Groups	RR (95% CI)	I-square
Change of Ejection Fraction (EF) from Baseline (%)*	RCT	1.64 (-1.08, 4.37)	71%
	Observational	-1.34 (-3.49, 0.81)	1%
All-cause Mortality	RCT	0.85 (0.78, 0.92)^	0%
	Observational	0.68 (0.52, 0.89)^	0%
Hospitalization	RCT	0.76 (0.52, 1.10)	87%
	Observational	1.33 (1.16, 1.53)^	0%

Safety Analysis

Table 4 shows the meta-analysis of adverse events between patients in ARNI group and patients in the control group. No significant difference was there between two groups in the risk of hyperkalemia (RR: 1.19, 95% CI: 0.84, 1.68) and acute kidney injury (RR: 0.91, 95% CI: 0.75, 1.11). However, risk of hypotension was significantly 1.51 times in patients receiving ARNI compared to the placebo group (RR: 1.51. 95% CI: 1.37, 1.65).

**Table 4:** Results of adverse events * Significant at p-value<0.05 RR: Risk ratio; CI: confidence interval

Outcome	RR (95% CI)	P-value	I-square
Hyperkalemia	1.19 (0.84, 1.68)	0.33	65%
Acute Kidney Injury	0.91 (0.75, 1.11)	0.35	35%
Hypotension	1.51 (1.37, 1.65)	0.001*	25%

Discussion

The present meta-analysis compared ARNI with ARB and ACEI, including more recently conducted clinical trials and retrospective studies, and found the superiority of ARNI in terms of all-cause mortality and cardiac death outcomes. However, treatment with ARNI was also associated with an increased risk of hypotension. No significant differences were reported between the two study groups in terms of change in EF. The study conducted by Nielsen et al. performed a pairwise meta-analysis, which included 39 studies [20], and also found a beneficial impact of ARNI compared to ACEI or ARB in terms of all-cause mortality and serious adverse events. Our study was unique in that we used recently conducted RCTs and retrospective studies to conduct a pooled meta-analysis.

One study showed that optimal execution of ARNI therapy was empirically estimated to prevent more than 28,000 deaths each year [21]. ARNI combines an ARB (valsartan) with a neprilysin inhibitor (sacubitril), which leads to the inhibition of both the renin-angiotensin-aldosterone system (RAAS) and the natriuretic peptide system (NPS). By inhibiting the RAAS, ARNI reduces vasoconstriction and sodium retention, while by inhibiting the NPS, it increases vasodilation and diuresis. This dual mechanism of action has been shown to improve clinical outcomes in patients with HFrEF [22]. The findings of our meta-analysis are consistent with the guidelines issued by the American College of Cardiology (ACC)/American Heart Association (AHA)/Heart Failure Society of America (HFSA), which recommend the use of ARNI as the first-line therapy for HFrEF [23]. Due to the beneficial impacts of ARNI, several guidelines recommend its use for the management of heart failure [7,24].

The present meta-analysis reported an increased risk of hypotension in patients receiving ARNI treatment, possibly due to the diuretic, natriuretic and vasodilatory effects of natriuretic peptides [25]. The increased risk of hypotension is not associated with an increased risk of acute kidney injury, as a lower risk of injury was reported in patients receiving ARNI. However, the difference was statistically insignificant. Findings of the PARADISE MI study showed no difference in renal impairment in patients receiving ARNI and ACEI [26].

It is proposed that any eligible patient with HFrEF should consider switching to ARNI. Additionally, it is crucial to improve education among physicians, emphasizing not just the initiation of therapy but also the need to increase the dosage to reach the target level. With the proven benefits of ARNI for HFrEF, insurance approval for ARNI is increasingly being granted [27]. However, the cost factor cannot be ignored, which unfortunately serves as a hindrance, particularly for uninsured patients who lack adequate access to healthcare and treatments. As we strived to assess the effectiveness of ARNI therapy for new indications such as HF with mid-range and preserved EF, we should not neglect the need to enhance its ability to reduce morbidity and mortality in HFrEF. This can be achieved by ensuring that those who have already benefited from ARNI therapy have continued access to it.

The present meta-analysis has certain limitations. Firstly, patient-level data were not available in a large number of studies. So, we were not able to analyze the effect of different patients’ characteristics on the impact of ARNI on outcomes in patients with HFrEF. Studies also differed by definition of certain endpoints, including acute kidney injury. Moreover, differences were there in the choice of the control group for studies included in the current meta-analysis. The majority of the studies included used enalapril (ACEI).

## Conclusions

In conclusion, this meta-analysis showed that treatment with ARNI was associated with a significantly lower risk of all-cause mortality and cardiovascular death compared to control groups. There was no significant difference between the two groups in terms of change of EF from baseline or hospitalization related to heart failure. However, the risk of hypotension was significantly higher in patients receiving ARNI. The study findings support the use of ARNI as first-line therapy for heart failure with reduced ejection fraction. Further studies are required to determine the optimal use of ARNI in heart failure management and to investigate the mechanisms underlying the increased risk of hypotension.
